# Comprehensive Medical Support in Complex Emergencies (CMSCE): pilot course review

**DOI:** 10.1186/s12992-022-00809-5

**Published:** 2022-04-12

**Authors:** John M Quinn, Trisha Jigar, Michael Reinwald, Percy S T Annan, Thomas Aapore, James M Wilson, Margaret Ellis Bourdeaux, Timo Ulrichs, Martin CM Bricknell, Alan Moore, Stefan Goebbels, Christian Haggenmiller

**Affiliations:** 1 Prague Center for Global Health Institute of Hygiene and Epidemiology First Faculty of Medicine, Charles University, Prague, Czechia; 2 First Faculty of Medicine, Institute of Hygiene and Epidemiology Prague Center for Global Health, Dhabalia Global Health Intern Charles University, Prague, Czechia; 3 German Armed Forces Course Director Kofi Annan International Peacekeeping Training Centre (KAIPTC), Teshie, Ghana; 4 37 Military Hospital Accra Ghana, Accra, Ghana; 5 M2 Medical Intelligence, Inc, Accra, Ghana; 6 Ghana Red Cross Society Ghana, Accra, Ghana; 7 Security and Global Health Project Belfer Center Harvard Kennedy School of Government Instructor, Department of Global Health and Social Change, Harvard Medical School, Boston, USA; 8 Institute for Research in International, Assistance Akkon University for Human Sciences, Berlin, Germany; 9 Health and Military Medicine, Conflict and Health Research Group School of Security Studies, King’s College London, K4L.10 Kings Building, WC2R 2LS London, UK; 10 FRICS Western Health & Social Care Trust Altnagelvin Hospital Londonderr y United, BT47 6SB Londonderry, UK; 11 United Nations Division of Healthcare Management And Occupational Safety and Health, New York, USA; 12 German Institute for Defence and Strategic Studies (GIDS) Research Coordinator Health Security Interface & CMSCE Chief Facilitator, Hamburg, Germany

**Keywords:** Complex Emergencies, United Nations, Emergency response, Health Security

## Abstract

Global threats to health and health security are growing. Fragile and failed states, armed groups, ungoverned spaces, outbreaks and potential unknown “Disease X” threats, antimicrobial resistance (AMR), hybrid and gray zone conflict all exacerbate complex medical emergencies. These growing threats increase preventable morbidity and mortality of the most vulnerable populations. In an effort to promote best practices, standardize responses, and prevent excess death and disability in these contexts, The Kofi Annan International Peacekeeping Training Centre (KAIPTC), with support from multiple international partners and a volunteer facilitator faculty, administered the pilot course for military and civilian health officers involved in U.N. peacekeeping missions entitled, “*Comprehensive Medical Support in Complex Emergencies (CMSCE 19)*.” This brief review paper provides a description of the process in designing and delivering an interdisciplinary course for providers and decision makers responding to complex emergencies. We conclude with best practices and next steps for course evolution.

## Introduction

Health care professionals responding to complex medical emergencies and humanitarian crises must be prepared to adapt best practices that maximize care, especially in impoverished environments [1]. State fragility and failure gives rise to armed groups, increases actors committing violence and reduces the capacity of government [2–4]. Political institutions and social organizations experience a reduced accountability with respect to a Responsibility to Protect (R2P) [5, 6]. The combined threats of conflict and disease hinder health security, democracy, economic growth, stability, human protection and peace. Climate change, environmental degradation, economic and financial instability may further exacerbate the negative effects of complex emergencies and humanitarian crises and disasters.

In both long standing and emergency missions, disaster, pandemic and outbreak response produce significant challenges to interoperability by stakeholders and actors and may lead to increases in preventable morbidity and mortality. Health providers from the civilian and military domains and actors providing medical support in disasters, crisis, war, and conflict are increasingly targets of violence [7, 8]. Cross-agency and cross-health responder communication during and after emergencies, and standing forums to exchange of lessons learned are vital to maximize success and ensure effective and efficient crisis response.

Complex emergencies witness a rise in mortality and morbidity among the population, either as a result of the direct effects of war or conflict, or indirectly through the increased prevalence of disease (i.e. malnutrition, transmission of communicable diseases); these are often the result of deliberate political strategies on the part of the parties to a conflict (military, armed groups, militia etc.) [9, 10].

Characteristics of complex emergencies include a large number of civilian victims, populations who are besieged or displaced, human suffering on a major scale; substantial international assistance is needed and the response goes beyond the mandate or capacity of any one agency; delivery of humanitarian assistance is impeded or prevented by parties to the conflict; high security risks for relief workers providing humanitarian assistance; and relief workers targeted by parties to the conflict [11, 12]. Complex emergencies require coordination between military and civilian entities.

Military and security forces face increasingly complex scenarios requiring crisis management that includes the use of medical and health capabilities. The civil-military interface requires not only evidenced-based practices and policy rooted in precedent, but also immediate information exchange. Civilian-military interoperability prove vital during complex medical emergencies - especially in areas of conflict [13]; Korram-Manesh, et al. 2019; [14]. For example, addressing the 2014-2015 polio and 2017-present day measles outbreak in Ukraine is complicated by the outbreak of hostilities. The war in Ukraine poses major challenges for access to the most vulnerable populations for vaccine administration and only the Ministry of Health with support of Defense forces supported mitigation of the health crisis [15].

## Basic Definitions

**Table:** 

**Term**	**Definition**
Disaster	A serious event that causes a systemic breakdown in the relationship between humans and their environment on a scale that requires extraordinary efforts to allow the community to cope, and often requires outside help and international aid [9, 16, 17].Disasters are divided into two major categories: those caused by natural phenomenon and those caused by humans.
Complex Emergency	A humanitarian crisis in a country, region orsociety where there is a total or considerable breakdown of authority resulting from internal or external conflict, and which requires an international response that goes beyond the capacity of any single agency and/or the ongoing UN country program.
Responsibility to Protect (R2P)	The responsibility to protect embodies a political commitment to end the worst forms of violence and persecution; it seeks to narrow the gap between Member States’ pre-existing obligations under international humanitarian and human rights law and the reality faced by populations at risk of genocide, war crimes, ethnic cleansing and crimesagainst humanity [18].

The Kofi Annan International Peacekeeping Training Centre (KAIPTC) is an internationally recognized training center and a Centre of Excellence (COE) of the Economic Community of West African States (ECOWAS). The Ghana Ministry of Defence (MoD) established the Kofi Annan International Peacekeeping Training Centre (KAIPTC) in 1998 and commissioned it in 2004. The purpose was to build upon and share Ghana’s five decades of internationally acclaimed experience and competence in peace operations with other states in the Economic Community of West African States (ECOWAS) region and the rest of Africa.

The Kofi Annan International Peacekeeping Training Centre (KAIPTC) provides an environment in which the specific challenges to standardize education and universal training in promotion of evidence-based practices in interagency coordination and collaboration can be shared with representatives of multiple institutions with global impact.

The KAIPTC serves in the research and training for conflict prevention, conflict management and conflict resolution and sustainable delivery of enhanced regional capacity building for peace support operations [19]. Courses taught at the KAIPTC include, peace and support operations, conflict management and peace and security studied. Masters and PhD programs are available. The KAIPTC is a magnet for nonpartisan, nonjudgmental best practices for peace and stability operations for the African Continent and the international community that promote peace.

## The Comprehensive Medical Support in Complex Emergencies (CMSCE) Course

The aim of this course was to promote an integrated health response though enhancing interoperability across civilian and military actors that respond to complex emergencies. The central theme was to promote and develop an understanding among multiple humanitarian, governmental and military actors. Key health challenges include a lack of access to health care, destroyed healthcare infrastructure, medical and disaster logistics and information sharing, lack of health workers and emerging epidemiological pressures. The course is focused on the operational principles that drive the response to disrupted health systems using civilian and military contributions to health, access to health and strengthening response throughout the disaster cycle. The course is designed to create discussions between participants that aim to improve understanding of the controversies and challenges associated with the civilian-military interface across all domains that meet on the humanitarian field of operations.

## Course Methodology

This course was not a didactic instructor to student format, but rather a facilitator with participant process. Specifically, a Collaborative Problem Based Learning (CPBL) approach facilitated open discussion and sharing of experience. Combining this with core skills and guidance, the environment allowed the emergence of a shared view juxtaposed to current best practices.

Evidence based decision making and practice is the anticipated outcome from CPBL. In this model that was deployed for this pilot course, participants’ interactions and contributions support success in the learning environment.

## The Pilot Course

The design of this course set out a one-week curriculum conducted at the KAIPTC to expose course participants to relevant and current skills, expertise and best practices in the domain of medical support and cooperation. The pilot course had specific emphasis on the African continent with lessons that can be transferred globally. This course was initiated by the German Institute for Defence and Strategic Studies (GIDS) and co-organized with the KAIPTC. The initial funding for this pilot course and the next course in 2020 is provided by the German government. To ensure sustainability, the goal is to invite multiple sponsoring nations and institutions for future courses.

The CMSCE courses’ main aims were to promote an integrated health response through enhanced interoperability across civilian and military actors in complex emergencies. This included value added from unofficial communication and networking across agencies, domains and countries supporting these best practices, especially partners and actors that face the greatest challenges in conflict.

## Setting

The CMSCE course took place in Accra Ghana at the KAIPTC from 11 to 15 November 2019. The training audience and target group for this novel pilot course included Senior Medical Officers (SMOs) and Force Medical Officers (FMOs) from the United Nations (UN), the African Union (AU), the Economic Community of West African States (ECOWAS), The North Atlantic Treaty Organization (NATO), the European Union (EU), Non-governmental organizations (NGOs). International civilian and military personnel preparing to serve on a myriad of missions, crisis and disasters in support of best practices.

The specific objectives of the course focused on the creation of an understanding of the definition of the complex environment of humanitarian support in conflict situations. This included the different agendas of the main civilian and military stakeholders in international crisis management. Emphasis throughout the curriculum focused on international guidelines, concepts and principles and their application to complex emergencies. Basic definitions focused on the complex relations and interdependence of health and human security. Discussion and debate were encouraged on the analysis of capacity and capability building programs that strengthen health systems in conflict and disaster, especially in affected regions that require joint health and security actors.

In addition, the course promoted the International Committee of the Red Cross and Red Crescent (ICRC) ‘Health in Danger’ project that highlight ethics, rights and responsibilities of healthcare staff. This included discussion on International Humanitarian Law (IHL), international human rights of caregivers and patient safety. Indeed, communities and all actors must address and reduce violence against healthcare workers. Finally, the main framework in disaster risk reduction, prevention and mitigation, including situational awareness, were presented.

## Participants

Participants were selected with attention to a balanced geographical, ethnic, gender and professional distribution. Participants followed the target groups listed above and included special invitation from 15 UN missions, national and international organizations, ministries, think tanks and centers for global health and professionals with an interest to learn about medical coordination and cooperation in key UN mission areas. There were over 47 applications received online through the KAIPTC Learning Management System (LMS). Of these 47 applicants, 17 were invited and 23 participants completed the course. Seven women participated.

## Highlighted Core Course Content

To only serve as a superficial review of some of the core topics addressed in the pilot course, this section highlights the central themes. The course timetable designed directed a ‘learning journey’ across one week as opposed to a didactic lecture series.

- Day 1 included a review of foundational concepts of health security, complex emergencies, and key actors involved in humanitarian assistance. In the opening session on ‘what is health’, perspectives on human security, national security and global security with consideration of the formal and informal actors in the health economy within a country were presented.
- Day 2 explored perspectives of organizational actors and stakeholders such as emergency humanitarian responders, public health, peacekeeping missions, and health security intelligence. The humanitarian charter, humanitarian imperative and international law were presented and reflected upon with respect to specific situations in military conflicts [20]. This section also highlighted medical ethics in conflict with discussion of the law of armed conflict and international humanitarian law with specific reference to the rights and duties of healthcare workers and ‘healthcare in danger.’

- Day 3 introduced challenges in civilian-military response to conflict, medical ethics in conflict, and included an offsite military hospital visit that included an interactive discussion of how to address mental health in complex emergencies for deployment. Organizational perspectives included the planning cycle in military medical operations with civilian actors as a mitigation of the outcomes of intentional violence between armed actors. One module of the course contained basic information about the ethical and legal backdrops of humanitarian aid.
- Days 4 the SPHERE^1^ concept and a scenario-based tabletop exercise (TTE) were introduced [21]. Principles and minimal standards of humanitarian aid in the sections of Water, Sanitation and Hygiene (WASH), food, shelter and medical aid were described in order to prepare the participants for the tabletop exercise (TTE) so they could practice referencing humanitarian standards as well as address a global health complex emergency. The TTE spanned 1.5 days and included a practical approach to SPHERE methodologies and encouraged out-of-the-box thinking in the approaches to complex emergencies.
- On day 5, the TTE scenario continued in the presentation of a complex humanitarian crisis with overlays of food insecurity and conflict that included an inject of a “Disease X” outbreak. The emphasis of the TTE was on collaboration among the various actors, understanding how to interpret indicator information as a vital component to situational awareness, and how to connect that information to preparedness and response. The TTE reflection and insights from participants on considerations going forward. The participants shared and reflected upon the concepts applied and knowledge gained over the week course and how they would apply core principles into their respective practice.

## Monitoring and Evaluation Outcomes and next steps

The CMSCE Pilot course was successfully conducted and participants and facilitators were both vital to the success. Based on feedback from participants and facilitators, and a facilitator roundtable review, many outcomes can be described. Going forward, the CMSCE will be offered in two separate courses: basic and advanced. The basic course will be foundational and will focus on principles and common guidelines and definitions found in the SPHERE handbook, multiple UN disaster and outbreak response guidance and other core definitions used for those who respond to complex emergencies. Owing to feedback about practical applications and healthcare under threat, this basic course will also include the very practical first aid at point of injury found in the UN guidelines and military medicine. These concepts found in the paradigm of tactical emergency / combat casualty care (TECC / TCCC) which focus on preventable causes of death from war related trauma.

The advanced course will be run as a workshop and will assume a foundational understanding of basic core concepts and will instead address current and emergent topics for subject matter experts and senior leadership responding to complex emergencies on a strategic level. The advanced workshop will have different topics each iteration and the curriculum will be designed and created in 2020.

Another outcome to maximize time during the course is to introduce a more robust online learning environment with open-source materials in the form of videos, articles, chapters, online quizzes and other academic materials. This eLearning component will be introduced through the existing KAIPTC Learning Management System (LMS). This eLearning component aims to present core definitions while course participants are still in their home country prior to arriving to KAIPTC and taking the live portion of the course.

## Conclusions

In summary, global health threats are increasing, and exacerbating factors are making complex medical emergencies ever more difficult to approach and apply best practices. In an effort to promote best practices, to standardize response and to mitigate preventable death and morbidity, the Kofi Annan International Peacekeeping Training Centre (KAIPTC) administered the pilot course entitled, “*Comprehensive Medical Support in Complex Emergencies (CMSCE 19)*.” The next courses to be offered will breakdown into a basic and an advanced level for practitioners.

This brief review paper describes the process of designing and delivering this interdisciplinary pilot course and sets forth a roadmap for the future.

## Data Availability

The datasets generated and/or analyzed during the current study are available in the KAIPTC repository.

## Footnotes

1 The Sphere Project and its Handbook are well known for introducing considerations of quality and accountability to humanitarian response
